# Feasibility and Clinical Outcomes of Robot-Assisted Sacrocolpopexy Using Autologous Round Ligament Grafts: A Novel Non-Mesh Surgical Approach for Pelvic Organ Prolapse

**DOI:** 10.3390/medicina61071242

**Published:** 2025-07-09

**Authors:** Shinichi Togami, Takashi Ushiwaka, Nozomi Furuzono, Yusuke Kobayashi, Chikako Nagata, Mika Fukuda, Mika Mizuno, Shintaro Yanazume, Hiroaki Kobayashi

**Affiliations:** 1 Department of Obstetrics and Gynecology, Faculty of Medicine, Kagoshima University, Kagoshima 890-8520, Japan; togami@m3.kufm.kagoshima-u.ac.jp (S.T.); takashi-u@kochi-u.ac.jp (T.U.); nozomi.fur051120@gmail.com (N.F.); ykobayashi@kufm.kagoshima-u.ac.jp (Y.K.); nagata1015.kag@gmail.com (C.N.); sakura10@m2.kufm.kagoshima-u.ac.jp (M.F.); mizunomizuno512@gmail.com (M.M.); s-yana@m3.kufm.kagoshima-u.ac.jp (S.Y.); 2 Department of Obstetrics and Gynecology, Kochi Medical School, Kochi University, Kochi 780-8520, Japan

**Keywords:** conversion to open surgery, pelvic organ prolapse, robotic surgical procedures, surgical mesh, urinary incontinence

## Abstract

*Background and Objectives:* To evaluate the feasibility and clinical outcomes of a novel non-mesh robot-assisted sacrocolpopexy (RSC) using autologous round ligament (ARL) grafts in patients with pelvic organ prolapse (POP). *Materials and Methods:* This retrospective study included 92 patients who underwent non-mesh RSC with ARL grafts at Kagoshima University Hospital between August 2020 and June 2024. All patients met the inclusion criteria for symptomatic POP-Q stage II or higher and elected to undergo non-mesh RSC. The procedures were performed using the da Vinci^®^ Xi or the hinotori™ Surgical Robot System. The clinical characteristics, operative data, complications, and recurrence rates were analyzed. *Results:* ARL harvesting was feasible in all patients, and the non-mesh RSC procedure was completed without conversion to open surgery or any intraoperative complications. The median operative time was 251 min, and the median blood loss was 30 mL. Postoperative complications of Clavien-Dindo grade ≥ 2 occurred in five patients (5%), all of whom developed pelvic infections. De novo stress urinary incontinence was observed in one patient (1%). POP recurrence occurred in seven patients (8%) during a median follow-up of 3 months (range, 3–18 months), all of whom presented with cystocele. Five patients underwent reoperation, and two were managed conservatively. All patients experienced postoperative symptomatic improvement. A higher BMI and advanced POP-Q stage were significant predictors of recurrence. *Conclusions:* This is the first report of non-mesh RSC using an ARL graft. The procedure is feasible and effective, avoids the use of synthetic mesh, and offers short-term outcomes comparable to those of mesh-based RSC. ARL-based RSC represents a promising alternative, especially for patients at risk of mesh-related complications. Long-term follow-up is required to confirm durability.

## 1. Introduction

Pelvic organ prolapse (POP) is characterized by the descent or herniation of pelvic organs such as the bladder, uterus, and rectum through the vaginal canal. It is prevalent among older women and can lead to urinary and defecatory dysfunction, dyspareunia, and a significant decline in quality of life (QOL) [1]. Sacrocolpopexy is widely recognized as the gold standard surgical treatment for POP, offering excellent long-term anatomical and functional outcomes [2]. While traditionally performed via laparotomy, sacrocolpoplexy is increasingly performed using minimally invasive approaches, such as laparoscopy and robot-assisted surgery.

Robot-assisted sacrocolpopexy (RSC) has gained popularity owing to its enhanced three-dimensional visualization and precise instrument control, which facilitate dissection and suturing in the deep pelvis. These advantages are expected to reduce surgeon fatigue and improve postoperative outcomes [3].

Conventional RSC typically involves the use of a synthetic mesh to suspend the vaginal apex to the anterior longitudinal ligament of the sacrum. However, mesh-related complications such as erosion, infection, and chronic pain have raised concerns regarding its safety. Furthermore, the use of synthetic mesh in patients with increased susceptibility to infection—such as those with diabetes mellitus or those undergoing chronic corticosteroid therapy—poses a heightened risk of mesh-related infections. This presents a significant clinical challenge because treatment options for these high-risk populations remain limited. Consequently, alternative techniques utilizing autologous tissue grafts, such as the autologous fascia lata, have been explored [4,5]. However, their clinical applications remain limited.

Diabetes mellitus (DM) has been shown to almost double the risk of mesh exposure (OR 1.87, 95% CI 1.35–2.57) [6]. This is partly due to an increased inflammatory response to the mesh in women with diabetes, which has been confirmed in murine models [7]. Understanding how diabetes influences prolapse recurrence is essential for optimizing care in populations where the disease is more prevalent.

To address the need for safer alternatives, we developed a novel non-mesh RSC technique using autologous round ligament (ARL) grafts. The round ligament is a naturally supportive pelvic tissue structure that is easily accessible during hysterectomy and carries no risk of foreign body reactions. Previously, a supracervical hysterectomy was often performed to reduce the risk of mesh erosion. However, the use of ARL grafts enables total hysterectomy with complete resection of the cervix while maintaining effective apical support without relying on synthetic mesh. To the best of our knowledge, no previous study has reported the use of ARL in sacrocolpopexy.

This study aimed to evaluate the clinical feasibility of non-mesh RSC using ARL by retrospectively analyzing surgical parameters, including operative time, blood loss, intraoperative and postoperative complications, and recurrence rates, in patients treated at our institution.

## 2. Materials and Methods

**Figure 1:**
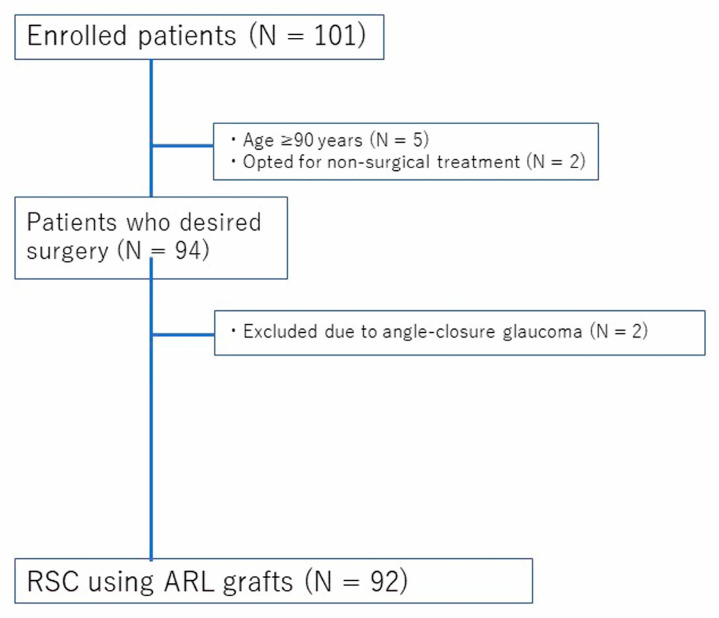
Flowchart of patient selection. A total of 101 patients were assessed for their eligibility. Seven patients were excluded due to age ≥ 90 years (*n* = 5) or preference for non-surgical treatment (*n* = 2). Among the 94 patients who desired surgery, 2 were excluded due to angle-closure glaucoma. Ultimately, 92 patients underwent robot-assisted sacrocolpopexy (RSC) with autologous round ligament (ARL) grafts.

This retrospective study included patients who underwent RSC at Kagoshima University Hospital between August 2020 and June 2024. The inclusion criteria were as follows: (1) POP-Q stage II or higher; (2) presence of prolapse-related symptoms, including pelvic pressure sensation or urinary/bowel dysfunction; (3) desire for surgical treatment; and (4) provision of written informed consent. Patients were excluded if they: (1) opted for conservative treatment; (2) were unable to provide informed consent due to severe psychiatric disorders; (3) were aged 90 years or older; or (4) had angle-closure glaucoma. These additional criteria were applied due to the increased anesthetic and perioperative risks associated with extreme age or contraindications to the Trendelenburg position, which is commonly used during robotic surgery. The patient selection process is illustrated in Figure 1. A total of 101 patients were initially assessed for their eligibility. After applying the exclusion criteria—including age ≥ 90 years, preference for non-surgical treatment, and the presence of angle-closure glaucoma—a total of 92 patients who underwent RSC using ARL grafts were included in the final analysis. This study was approved by the Institutional Review Board of Kagoshima University Hospital (IRB No.200251), and all participants provided written informed consent. Clinical and surgical data were extracted from the electronic medical records.

### 2.1. Surgical Technique

All procedures were performed using either the da Vinci^®^ Xi (Intuitive Surgical, Sunnyvale, CA, USA) or the hinotori™ Surgical Robot System (Medicaroid Corporation, Kobe, Japan). As part of the procedure, all patients underwent total hysterectomy with or without bilateral salpingo-oophorectomy. The procedures were performed by four gynecologic oncologists, two of whom were board-certified endoscopic surgeons. All participating surgeons were highly experienced and well-trained in robot-assisted gynecologic surgery.

**Figure 2:**
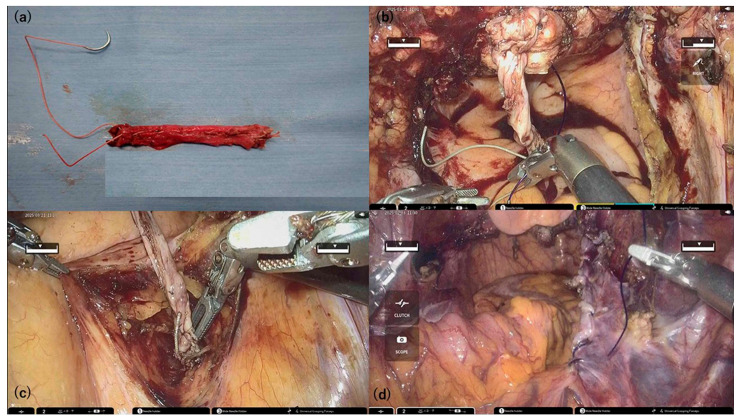
Surgical technique of non-mesh robotic sacrocolpopexy using autologous round ligament (ARL). (**a**) Two harvested round ligaments were aligned in series and reinforced with 2-0 Ethibond^®^ (Johnson & Johnson Ethicon, Cincinnati, OH, USA) sutures to construct the ARL graft. (**b**) Following total hysterectomy and vaginal cuff closure, the ARL graft is secured at point Aa on the anterior vaginal wall. (**c**) The opposite end of the ARL graft is anchored to the anterior longitudinal ligament at the sacral promontory. (**d**) The pelvic peritoneum is closed with continuous sutures, covering the ARL graft retroperitoneal space.

1. Both round ligaments were harvested at their maximum available length and extracted through the trocar for extracorporeal preparation. The two ligaments were aligned end-to-end, reinforced by folding, and sutured using 2-0 Ethibond^®^ thread to create an ARL graft. The resulting graft was approximately 7–9 cm long (Figure 2a).
2. A total of 1 mL of Indigo Carmine was injected transvaginally at point Aa to assist with anterior compartment dissection by delineating the bladder border. This technique enhances anatomical clarity and facilitates precise graft placement, especially in cases of severe pelvic organ prolapse.
3. Following vaginal cuff closure, one end of the ARL was fixed to the anterior vaginal wall at point Aa using blue dye as a landmark. The bladder-vaginal septum was plicated bilaterally with STRATAFIX™ Spiral PDS Plus (Ethicon Inc., Cincinnati, OH, USA) to reinforce the ARL (Figure 2b).
4. The right anterior surface of the sacrum was exposed, and the opposite end of the ARL was fixed to the anterior longitudinal ligament, elevating the vaginal apex (Figure 2c).
5. The central pelvic peritoneum was closed to position the ARL retroperitoneally (Figure 2d).
6. A pelvic drain was placed in the cul-de-sac, and adhesion barriers were applied before completing the robotic surgery.
7. Perineorrhaphy was performed via the vaginal route, and posterior colporrhaphy was performed as needed, depending on the degree of rectocele.

Bilateral ARL grafts were used because a unilateral round ligament typically lacks sufficient length to reach the sacral promontory while maintaining appropriate tension. The use of both ligaments ensures adequate graft length and anatomical support, which are essential for durable pelvic floor reconstruction.

A right-sided approach was used in all cases to expose the sacral promontory. Additionally, perineorrhaphy was routinely performed because most patients with recurrent or advanced POP presented significant perineal laxity. This step was incorporated to improve posterior support and enhance the long-term durability of the repair.

Postoperatively, patients were not instructed to perform Kegel exercises or any other specific pelvic floor training. Instead, they were uniformly advised to avoid behaviors that increase intra-abdominal pressure, such as heavy lifting, coughing without support, or straining during defecation, especially during the first few weeks after surgery.

### 2.2. Evaluation Parameters

The following parameters were assessed in this study: patient demographics and clinical background (including age, body mass index [BMI], parity, history of prior abdominal surgery, DM, POP-Q stage, and predominant prolapsed compartment), surgical factors (including type of robotic platform used, total operative time, console time, estimated blood loss, length of hospital stay, conversion to open surgery, and intraoperative and postoperative complications), and postoperative outcomes (including recurrence of pelvic organ prolapse and need for reoperation). Comorbid conditions were reviewed, and diabetes mellitus (DM) was recorded as a relevant accompanying disease due to its potential influence on mesh-free prolapse repair outcomes. Other systemic diseases were not uniformly documented and, therefore, not included in the analysis. Intraoperative complications were defined as any unintended events or adverse outcomes that occurred during surgery, from the initial incision to wound closure. These included organ injury, hemorrhage, anesthetic complications, and technical difficulties such as equipment malfunction. Postoperative complications were defined as adverse events arising after the completion of surgery, typically within the same hospitalization period or within 30 days postoperatively. These included surgical site infections, thromboembolic events, urinary retention, postoperative hemorrhage, and ileus. Recurrence was defined as the presence of POP-Q stage II or higher at the vaginal apex, bladder, or rectum or the need for pessary insertion or reoperation. Postoperative evaluation included symptom assessment and pelvic examination at 1 and 3 months. Subjective improvement in pelvic organ prolapse (POP) symptoms was assessed during postoperative outpatient visits based on patient-reported resolution of vaginal bulging, pelvic pressure, or other prolapse-related complaints. Validated questionnaires were not used. Objective anatomical success was defined as the postoperative location of the vaginal cuff being more than 5 cm above the hymenal ring (POP-Q point C ≤ −5 cm) following hysterectomy. After 3 months, the patients were instructed to return for follow-up in the event of recurrent symptoms or complications.

### 2.3. Statistical Analysis

All statistical analyses were performed using the JMP software (version 14.0; SAS Institute Inc., Cary, NC, USA). Continuous variables were analyzed using the Wilcoxon rank-sum test and are reported as medians with interquartile ranges. Categorical variables were compared using the chi-square test or Fisher’s exact test, as appropriate.

A multivariate logistic regression model was constructed to identify independent predictors of recurrence. The variables included in the model—age, body mass index (BMI), parity, diabetes mellitus (DM), POP-Q stage, and type of robotic system—were selected based on clinical relevance and prior literature. The type of robotic system (da Vinci^®^ Xi or hinotori™) was included as a background variable to account for differences in the surgical platforms, although no comparative analysis was intended. Odds ratios (ORs) with 95% confidence intervals (CIs) were calculated, and a *p*-value < 0.05 was considered statistically significant.

No formal adjustments for multiple comparisons were applied, as the primary aim of the analysis was to explore potential predictors of recurrence rather than to conduct confirmatory hypothesis testing across multiple subgroups.

## 3. Results

Graft harvesting using the round ligament was feasible in all 92 patients, enabling non-mesh RSC with ARL reconstruction. All patients reported symptomatic improvement in POP after surgery.

Table 1 summarizes the clinical characteristics of the 92 patients. The median age was 68 years (range, 40–89 years), and the median BMI was 25.0 kg/m^2^ (range, 18.9–38.4). The parity distribution was as follows: one delivery (six patients, 7%), two deliveries (40 patients, 43%), three deliveries (30 patients, 33%), and four or more deliveries (16 patients, 17%). DM was present in 26 (28 %) patients in the study cohort. Approximately one-third of the patients (29 patients, 32%) had undergone abdominal surgery. According to the POP-Q classification, 24 patients (26%) had stage II prolapse, 49 patients (53%) had stage III, and 19 patients (21%) had stage IV.

Regarding prolapsed organs, the majority (56 patients, 61%) exhibited both uterine and bladder prolapse, followed by combined prolapse of the uterus, bladder, and rectum (17 patients, 18%). Uterine prolapse alone and bladder prolapse alone were observed in nine patients each (10%), and one patient (1%) had uterine and rectal prolapse.

Table 2 presents the surgical characteristics of the 92 patients. The da Vinci^®^ Xi and hinotori™ SRS systems were used in 72% and 28% of cases, respectively. The median operative and console/cockpit times were 251 (123–612) min and 193 (93–430) min, respectively. The median blood loss and hospital stay were 30 mL (range, 1–160 mL) and 6 days (range, 4–18 days), respectively. None of the patients required conversion to open surgery, and no intraoperative complications were observed. Postoperative complications of Clavien–Dindo grade ≥ 2 were observed in five patients (5%), all of whom developed pelvic inflammatory disease (PID), diagnosed based on clinical symptoms such as lower abdominal pain and fever, and confirmed by imaging findings. All patients were successfully treated with intravenous antibiotics. One patient (1%) experienced de novo stress urinary incontinence (SUI), and recurrence occurred in seven patients (8%)—all presenting with cystoceles—during a median follow-up of 3 months (range, 3–18 months). The median follow-up period was 3 months. Five underwent reoperation (two sacrospinous ligament fixations, two colpocleisis procedures, and one anterior colporrhaphy), while two were managed conservatively with pessary insertion. These results indicate that non-mesh RSC using autologous round ligament grafts is feasible and safe, with low rates of complications and recurrence, even in a diverse population with varying degrees of prolapse and surgical history.

Multivariate logistic regression analysis was performed to identify the independent predictors of recurrence following robot-assisted sacrocolpopexy (RSC) using autologous round ligament (ARL) grafts (Table 3). Among the analyzed variables, a higher body mass index (BMI) and advanced POP-Q stage (Stage IV) were significantly associated with an increased risk of recurrence. Specifically, each unit increase in BMI was associated with a 1.28-fold higher odds of recurrence (OR: 1.276, 95% CI: 1.037–1.644, *p* = 0.0217). Additionally, patients with POP-Q stage IV had a markedly higher risk compared to those with Stage II or III (OR = 20.200, 95% CI: 2.848–214.059, *p* = 0.0026).

Other variables, such as age, parity (1 or 2 vs. ≥3), presence of diabetes mellitus (DM), and type of robotic surgical system (da Vinci^®^ Xi vs. hinotori™), were not statistically significant predictors. Notably, while DM had a relatively high odds ratio (OR = 5.236), the wide confidence interval (0.609–122.715) and *p*-value of 0.141 indicate an insufficient statistical power.

These findings suggest that preoperative BMI and the severity of prolapse (POP-Q stage) are important considerations when evaluating the recurrence risk in patients undergoing non-mesh RSC using ARL.

## 4. Discussion

To the best of our knowledge, this study is the first to report non-mesh RSC using autologous round ligament (ARL) grafts. Among the 92 patients, ARL graft creation was feasible and led to symptomatic improvement in POP without conversion to open surgery or intraoperative complications. Although postoperative complications (Clavien-Dindo grade ≥ 2) occurred in five cases (5%) and cystocele recurrence in seven cases (8%), these findings suggest that ARL-based non-mesh RSC is a viable surgical approach.

Common concerns regarding mesh-based RSC include erosion, infection, and mesh exposure. Several reports have suggested biological alternatives, such as the autologous fascia lata. Patel et al. [8] performed sacrocolpopexy using autologous fascia lata in 34 patients, reporting symptomatic improvement in all cases, suggesting its suitability for patients seeking to avoid synthetic meshes. Egbe et al. [9] described the use of autologous umbilical ligament grafts for robotic sacrocolpopexy, highlighting their potential to mitigate mesh-related risks. The fascia lata provides favorable tensile strength and histological compatibility as an autologous graft; however, its harvesting requires a thigh incision, which may cause postoperative pain and compromise the minimally invasive advantage of robotic surgery. In contrast, ARL grafts utilize round ligaments, which are typically excised during hysterectomy, avoiding the need for an additional incision, thereby reducing surgical invasiveness. The ARL technique is applicable in patients with a uterus, and its primary contraindication is the presence of uterine malignancy, making preoperative exclusion of malignancy essential. In this study, ARL-based non-mesh RSC was successfully performed in 92 patients, all of whom reported postoperative improvement in POP. Unlike mesh-based RSC, this approach also facilitates total hysterectomy and, when indicated, bilateral salpingo-oophorectomy without cervical retention, providing an additional oncological advantage.

Previous studies have reported similar recurrence rates for laparoscopic sacrocolpopexy (LSC) and RSC (10.8% and 9.2%, respectively) [10], while others have shown higher recurrence rates with RSC (25.9%) than with LSC (7.5%) [11]. Reported reoperation rates after RSC range from 4% to 9.2% [11,12,13]. In this study, the recurrence rate was 8%, and the reoperation rate was 5%, comparable to those of mesh-based RSC. However, all recurrences involved cystoceles, suggesting that additional anterior colporrhaphy may be necessary for patients with severe preoperative anterior compartment prolapse. Furthermore, we observed that a higher BMI and advanced preoperative POP-Q stage were significantly associated with an increased risk of POP recurrence following RSC using ARL grafts. These findings are consistent with those of previously published studies identifying both elevated BMI and advanced prolapse stage as important predictors of surgical failure in POP repair.

Friedman et al. [14] conducted a comprehensive meta-analysis and reported that advanced preoperative prolapse stage (stage III–IV) was associated with more than a twofold increased risk of recurrence following reconstructive surgery (OR 2.11, *p* < 0.001). Similarly, a higher BMI has been identified as a modest but statistically significant risk factor for recurrence, possibly due to chronically elevated intra-abdominal pressure exerting mechanical strain on pelvic floor support structures.

In a retrospective study by Eckhardt et al. [15], women with diabetes undergoing RSC exhibited both higher BMI and more advanced baseline POP stages, and multivariate analysis revealed that BMI significantly increased the hazard for both anterior vaginal wall prolapse recurrence and composite failure (HR 1.14 and 1.07, respectively). This reinforces the concept that patient-related factors, such as obesity and comorbid conditions, may compromise surgical durability, even in technically successful procedures.

The association between a higher POP-Q stage and recurrence may reflect underlying pelvic floor dysfunction or tissue integrity issues that are not fully addressed by surgical correction. As noted in the systematic review by Oh et al. [16], the long-term durability of sacrocolpopexy, including robotic approaches, may vary based on preoperative anatomical severity and patient characteristics.

In addition to BMI and POP-Q stage, Chang et al. [17] identified other important risk factors for recurrence after sacrocolpopexy. In their large retrospective cohort study with long-term follow-up, a preoperative genital hiatus size ≥ 4 cm was independently associated with increased odds of composite failure (adjusted OR 1.95, 95% CI 1.18–3.25). Furthermore, the need for concurrent anterior colporrhaphy was associated with a higher recurrence risk (adjusted OR 2.11), whereas concomitant posterior colporrhaphy was protective (adjusted OR 0.62). These findings suggest that anatomical factors related to the levator ani hiatus dimensions and the integrity of the anterior and posterior compartment support may substantially influence surgical outcomes.

Together, these results underscore the multifactorial nature of POP recurrence and highlight the importance of a comprehensive preoperative assessment, including the evaluation of genital hiatus size and compartment-specific prolapse severity. Individualized perioperative strategies, such as performing posterior repair when indicated, managing anterior defects cautiously, and addressing modifiable risk factors like obesity, may help improve long-term surgical durability, particularly in high-risk populations.

RSC is associated with shorter operative times, reduced blood loss, and lower conversion rates than LSC [18,19,20]. Capmas et al. [21] reported a conversion rate of 1.33% in 2777 RSC cases, significantly lower than the 7.14% observed in LSC. In comparison, a recent retrospective study reported a mean operative time of less than 200 min for robot-assisted sacrocolpopexy using synthetic mesh grafts [16], suggesting that our relatively longer operative time may be attributed to the use of autologous grafts and concurrent procedures, such as total hysterectomy, including the cervix. Nevertheless, minimal blood loss and lack of conversion support the safety of this procedure.

The post-hysterectomy surgical site infection (SSI) rate is generally 4.5% [22]. In this study, pelvic infections (Clavien-Dindo grade ≥ 2) occurred in five patients (5%), with no mesh-related complications such as erosion or spinal discitis. The absence of foreign materials likely contributed to the favorable safety profiles. The procedures utilized the da Vinci^®^ Xi and hinotori™ SRS systems. Reports have also indicated the feasibility of other emerging robotic platforms, such as the HUGO™ RAS and da Vinci^®^ single-site systems [23,24], suggesting a growing role for newer robotic systems in RSC.

The primary limitation of this study was its relatively short follow-up period of 3 months. Although the early outcomes demonstrated favorable feasibility and safety, long-term efficacy—including anatomical durability, recurrence rates, and graft-related complications—could not be adequately evaluated within this timeframe. Previous studies on pelvic organ prolapse surgery have emphasized the importance of ≥12-month follow-up periods to assess surgical durability. Therefore, the findings of the present study should be interpreted with caution, and future prospective studies with extended follow-ups are warranted to confirm the long-term efficacy and safety of this novel non-mesh approach.

Another potential limitation is selection bias. Patients included in this study consented to undergo a novel surgical technique, and those who opted for conservative management, were ≥90 years of age, or had contraindications to the Trendelenburg position (such as angle-closure glaucoma) were excluded. However, these individuals are typically not candidates for mesh-based RSC in standard clinical practice either. Therefore, we believe that the study population appropriately represents the patient cohort eligible for surgical intervention in real-world settings. Nevertheless, this may affect the generalizability of the findings and interpretation of recurrence rates.

Another limitation is the lack of standardized assessment tools for postoperative quality of life (QOL). Although patients were queried regarding symptoms such as vaginal bulging and urinary dysfunction, validated questionnaires such as the Pelvic Floor Distress Inventory (PFDI-20) or Pelvic Floor Impact Questionnaire (PFIQ-7) were not employed. Future research should incorporate these QOL instruments to ensure a comprehensive evaluation of patient-reported outcomes.

Additionally, although the use of autologous grafts without synthetic mesh may reduce material costs and avoid mesh-related complications, this study did not include a formal cost-effectiveness analysis. Further economic evaluations are needed to determine the financial impact of this novel surgical approach.

In our technique, a total hysterectomy was performed instead of a subtotal hysterectomy to reduce the risk of future cervical diseases and ensure anatomical durability. Preoperative transvaginal ultrasonography was routinely performed in all patients to evaluate the uterine cavity and endometrial thickness, ensuring that no suspicious intrauterine pathology was present prior to sacrocolpopexy. Ultrasound remains a valuable noninvasive tool for assessing uterine abnormalities and excluding conditions such as endometrial hyperplasia or polyps, especially in postmenopausal women undergoing pelvic reconstructive surgery [25]. A meta-analysis reported that concomitant hysterectomy was not associated with an increased risk of mesh-related complications, and there were no significant differences in recurrence or reoperation rates [26]. One advantage of this approach is the elimination of potential cervical pathologies, which is particularly beneficial for older women or those with limited access to regular gynecologic screening. Cervical elongation is also common in patients with POP, and if not adequately addressed, it may contribute to recurrence. Removing the cervix at the appropriate anatomical level helps prevent this potential failure point and supports long-term surgical success. Additionally, retaining the cervix poses a risk of future malignancies in the residual cervical tissue. Large retrospective studies evaluating POP repairs have reported the incidence of occult gynecologic malignancies at the time of surgery to range from 0.26% to 2.6% [27,28,29]. In cases where a mesh is present, such malignancies can pose significant treatment challenges. Therefore, total hysterectomy not only enhances anatomical correction but also mitigates oncologic risks, making it a particularly advantageous strategy for mesh-free robotic sacrocolpopexy.

## 5. Conclusions

In conclusion, this is the first study to report non-mesh RSC using an autologous ARL graft. The absence of a synthetic mesh enables total hysterectomy and eliminates the invasiveness associated with fascia lata harvesting. The short-term outcomes were comparable to those of mesh-based RSC, and continued case accumulation and long-term follow-up are warranted to further validate this technique.

## Figures and Tables

**Table 1:** Clinical characteristics of 92 patients who underwent robotic sacrocolpopexy.

	Patients (*n* = 92)
Median age (years)	68 (40–89)
Median BMI (kg/m^2^)	25 (18.9–38.4)
Parity	
1	6 (7%)
2	40 (43%)
3	30 (33%)
≤4	16 (17%)
History of abdominal surgery	
No	63 (68%)
Yes	29 (32%)
DM	
Absent	66 (72%)
Present	26 (28%)
POP-Q	
II	24 (26%)
III	49 (53%)
IV	19 (21%)
Prolapsed organ	
Uterus	9 (10%)
Bladder	9 (10%)
Uterus, bladder	56 (61%)
Uterus, rectum	1 (1%)
Uterus, bladder, rectum	17 (18%)

BMI, body mass index, DM, diabetes mellitus. All patients had type 2 diabetes mellitus. POP-Q: pelvic organ prolapse quantification.

**Table 2:** Surgical characteristics of 92 patients who underwent robotic sacrocolpopexy.

	Patients (*n* = 92)
Type of robotic system	
da Vinci^®^ Xi	66 (72%)
hinotori™ Surgical Robot System	26 (28%)
Median operation time (min)	251 (range, 123–612)
Median cockpit/console time (min)	193 (range, 93–430)
Median blood loss (mL)	30 (range, 1–160)
Median length of hospital stays (days)	6 (range, 4–18)
Conversion to open surgery	
No	92 (100%)
Yes	0 (0%)
Intraoperative complications	
No	92 (100%)
Yes	0 (0%)
Postoperative complications	
No	87 (95%)
Yes	5 (5%)
Recurrence	
No	85 (92%)
Yes	7 (8%)
De novo SUI	
No	91 (99%)
Yes	1 (1%)

De novo SUI: de novo stress urinary incontinence.

**Table 3:** Multivariate Logistic Regression Analysis of Predictors of Recurrence Following Robot-Assisted Sacrocolpopexy (RSC) Using Autologous Round Ligament (ARL) Grafts (*n* = 92).

Variable	Odds Ratio (OR)	95% CI for OR (Lower–Upper)	*p*-Value
Age (per year increase)	1.039	0.958–1.141	0.359
BMI	1.276	1.037–1.644	0.0217
Parity (1 or 2 vs. ≥3)	0.693	0.098–4.519	0.695
DM (negative vs. positive)	5.236	0.609–122.715	0.141
POP-Q (II or III vs. IV)	20.200	2.848–214.059	0.0026
Type of robotic system (da Vinci^®^ Xi vs. hinotori™ Surgical Robot System)	0.170	0.005–1.945	0.169

BMI, body mass index; DM, diabetes mellitus; POP-Q, pelvic organ prolapse quantification.

## Data Availability

The original contributions presented in this study are included in the article. Further inquiries can be directed to the corresponding author.

## References

[1] Martin B.S., Markowitz M.A., Myers E.R., Lundsberg L.S., Ringel N. (2024). Estimated national cost of pelvic organ prolapse surgery in the United States. Obs. Gynecol..

[2] Nygaard I., Brubaker L., Zyczynski H.M., Cundiff G., Richter H., Gantz M., Fine P., Menefee S., Ridgeway B., Visco A. (2013). Long-term outcomes following abdominal sacrocolpopexy for pelvic organ prolapse. JAMA.

[3] Tarr M.E., Brancato S.J., Cunkelman J.A., Polcari A., Nutter B., Kenton K. (2015). Comparison of postural ergonomics between laparoscopic and robotic sacrocolpopexy: A pilot study. J. Minim. Invasive Gynecol..

[4] Damiani G.R., Villa M., Falcicchio G., Cesana C., Malvasi A., Picardi N., Vergottini G., Piero P., Dellino M., Loizzi V. (2023). Robotic sacrocolpopexy with autologous fascia Lata: A case series. Gynecol. Minim. Invasive Ther..

[5] Scott V.C.S., Oliver J.L., Raz S., Kim J.H. (2019). Robot-assisted laparoscopic sacrocolpopexy with autologous fascia lata: Technique and initial outcomes. Int. Urogynecol. J..

[6] Deng T., Liao B., Luo D., Shen H., Wang K. (2016). Risk factors for mesh erosion after female pelvic floor reconstructive surgery: A systematic review and meta-analysis. BJU Int..

[7] Liang R., Fisk A., King G., Meyn L., Xiao X., Moalli P. (2022). Characterization of vaginal immune response to a polypropylene mesh: Diabetic vs. normoglycemic conditions. Acta. Biomater..

[8] Patel S., Chaus F.M., Funk J.T., Twiss C.O. (2022). Total autologous fascia Lata sacrocolpopexy for treatment of pelvic organ prolapse: Experience in thirty-four patients. Urology.

[9] Egbe A., Issa R., Walker R., Tay A., Seth J., Rashid T. (2023). Robotic sacrocolpopexy with medial umbilical ligament autologous graft to treat neovaginal prolapse in a transgender woman. Urol. Case Rep..

[10] Zhang Y., Jiang X., Mao M., Bai J., Tian Y., Sun W., Guo R. (2025). No difference in prolapse recurrence rates between laparoscopic and robotic-assisted sacrocolpopexy: A long-term comparison. J. Minim. Invasive Gynecol..

[11] Lallemant M., Tresch C., Puyraveau M., Delplanque S., Cosson M., Ramanah R. (2021). Evaluating the morbidity and long-term efficacy of laparoscopic sacrocolpopexy with and without robotic assistance for pelvic organ prolapse. J. Robot. Surg..

[12] Culligan P.J., Lewis C., Priestley J., Mushonga N. (2020). Long-term outcomes of robotic-assisted laparoscopic sacrocolpopexy using lightweight Y-mesh. Female Pelvic Med. Reconstr. Surg..

[13] Guérin S., Suzman E., Alhalabi F., Lutz K., Zimmern P. (2024). Very long-term outcomes of robotic mesh sacrocolpopexy for pelvic organ prolapse repair. J. Robot. Surg..

[14] Friedman T., Eslick G.D., Dietz H.P. (2018). Risk factors for prolapse recurrence: Systematic review and meta-analysis. Int. Urogynecol. J..

[15] Eckhardt S.E., Laus K., DeAndrade S., Lee J., Nguyen J. (2023). The impact of diabetes mellitus on pelvic organ prolapse recurrence after robotic sacrocolpopexy. Int. Urogynecol. J..

[16] Oh S., Shin J.H. (2023). Outcomes of robotic sacrocolpopexy. Obstet. Gynecol. Sci..

[17] Chang O.H., Davidson E.R., Thomas T.N., Paraiso M.F.R., Ferrando C.A. (2021). Predictors for Pelvic Organ Prolapse Recurrence After Sacrocolpopexy: A Matched Case-Control Study. Female Pelvic Med. Reconstr. Surg..

[18] Evangelopoulos N., Nessi A., Achtari C. (2024). Minimally invasive sacrocolpopexy: Efficiency of robotic assistance compared to standard laparoscopy. J. Robot. Surg..

[19] Chang C.L., Chen C.H., Yang S.S.D., Chang S.J. (2022). An updated systematic review and network meta-analysis comparing open, laparoscopic and robotic-assisted sacrocolpopexy for managing pelvic organ prolapse. J. Robot. Surg..

[20] Chang C.L., Chen C.H., Chang S.J. (2022). Comparing the outcomes and effectiveness of robotic-assisted sacrocolpopexy and laparoscopic sacrocolpopexy in the treatment of pelvic organ prolapse. Int. Urogynecol. J..

[21] Capmas P., Suarthana E., Larouche M. (2021). Conversion rate of laparoscopic or robotic to open sacrocolpopexy: Are there associated factors and complications?. Int. Urogynecol. J..

[22] Andiman S.E., Xu X., Boyce J.M., Ludwig E.M., Rillstone H.R.W., Desai V.B., Fan L.L. (2018). Decreased surgical site infection rate in hysterectomy: Effect of a gynecology-specific bundle. Obs. Gynecol..

[23] Mueller M.G., Ashmore S., Collins S., Lewicky-Gaupp C., Kenton K. (2024). Single-port robotic sacrocolpopexy: Description of an advanced minimally invasive approach and review of the relevant literature. Int. Urogynecol. J..

[24] Panico G., Vacca L., Campagna G., Caramazza D., Mastrovito S., Lombisani A., Ercoli A., Scambia G. (2023). The first 60 cases of robotic sacrocolpopexy with the novel HUGO RAS system: Feasibility, setting and perioperative outcomes. Front. Surg..

[25] Nguyen P.N., Nguyen V.T. (2023). Additional value of Doppler ultrasound to B-mode ultrasound in assessing for uterine intracavitary pathologies among perimenopausal and postmenopausal bleeding women: A multicentre prospective observational study in Vietnam. J. Ultrasound.

[26] Tius V., Arcieri M., Taliento C., Pellecchia G., Capobianco G., Simoncini T., Giovanni P., Caramazza D., Campagna G., Driul L. (2025). Laparoscopic sacrocolpopexy with concurrent hysterectomy or uterine preservation: A metanalysis and systematic review. Int. J. Gynaecol. Obstet..

[27] Andy U.U., Nosti P.A., Kane S., Deeb M., Brucker B.M., Lowenstein L. (2014). Incidence of unanticipated uterine pathology at the time of minimally invasive abdominal sacrocolpopexy. J. Minim. Invasive Gynecol..

[28] Ramm O., Gleason J.L., Segal S., Antosh D.D., Kenton K.S. (2012). Utility of preoperative endometrial assessment in asymptomatic women undergoing hysterectomy for pelvic floor dysfunction. Int. Urogynecol. J..

[29] Renganathan A., Edwards R., Duckett J.R.A. (2010). Uterus conserving prolapse surgery—What is the chance of missing a malignancy?. Int. Urogynecol. J..

